# Decoding glycosylation in cardiovascular diseases: mechanisms, biomarkers, and therapeutic opportunities

**DOI:** 10.3389/fphar.2025.1570158

**Published:** 2025-05-19

**Authors:** Gang Li, Kaidi Ren, Yage Jin, Yang Yang, Yi Luan

**Affiliations:** ^1^ Department of Cardiology, The First Affiliated Hospital of Zhengzhou University, Zhengzhou, China; ^2^ Department of Pharmacy, The First Affiliated Hospital of Zhengzhou University, Zhengzhou, China; ^3^ Clinical Systems Biology Laboratories, The First Affiliated Hospital of Zhengzhou University, Zhengzhou, China

**Keywords:** protein glycosylation, O-GlcNAcylation, cardiovascular diseases, OGT, OGA

## Abstract

Protein glycosylation, particularly O-GlcNAcylation, is a critical post-translational modification (PTM) that regulates cardiac and vascular functions by modulating protein stability, localization, and interactions. Dysregulated glycosylation is generally believed as a key driver in the pathogenesis of cardiovascular diseases (CVDs), contributing to adverse cardiac remodeling, mitochondrial dysfunction, metabolic dysregulation, and vascular inflammation. This review highlights the mechanistic roles of glycosylation in CVD progression, including its regulation of cardiac remodeling, mitochondrial dysfunction, and vascular inflammation. This study explored the dual role of O-GlcNAcylation in acute protection and chronic injury, emphasizing its potential as a biomarker for early diagnosis and risk stratification. Therapeutic strategies targeting glycosylation pathways, particularly O-GlcNAc transferase (OGT), and O-GlcNAcase (OGA), hold promise for addressing myocardial ischemia-reperfusion injury, diabetic cardiomyopathy, and atherosclerosis. Advances in glycosylation profiling and interdisciplinary collaboration are essential to overcome challenges such as tissue specificity and off-target effects, advancing precision cardiovascular medicine.

## Introduction

Cardiovascular disease (CVD) remains a prominent risk factor for human death and disability worldwide. As depicted from data from 1999–2019, the number of people suffering from CVD almost doubled, increasing from 271 million to 523 million, and the mortality arising from 12.1 million to 18.6 million (Zhou et al., 2023; Beadle et al., 2015; Park et al., 2016). Despite recent advances in deciphering the pathogenesis of CVD, it is still insufficient to fully address and intervene in cardiovascular disorders. Therefore, the identification of novel diagnostic methods and the generation of appropriate therapies are urgently needed for the risk prediction of future CVD events. Among multiple research insights, glycomics is a reliable approach for proposing innovative strategies and offering novel treatment targets.

Protein post-translational modifications (PTMs) cause rapid alterations in protein function, location, turnover and crosstalk, which greatly increase the variety of proteome (Smith and White, 2014). Among these modifications, protein glycosylation makes up approximately 20%–50% of proteins glycosylated, making it one of the most abundant protein modifications (Gajjala et al., 2015; Stastna, 2024). In protein glycosylation, carbohydrates are linked to certain amino acids. In mammals, nine different amino acids can initially be attached to nine different monosaccharides (Chatham and R, 2024). Carbohydrate structures are also composed of monosaccharides and oligosaccharides, called glycans, which can also be further modified by other protein modifications, such as phosphorylation, acetylation, and sulfation, resulting in a more diversified glycan structure (Lawler, 2016). These diverse structures generate a large amount of structural variation that is impossible with amino acids or nucleic acids alone (Biros et al., 2015). Most glycans are located on the outer membrane of cells and produce diverse macromolecules. Simple and highly dynamic glycans can also be found throughout cells, where they act as regulatory modules (Hoshi et al., 2024).

**FIGURE 1:**
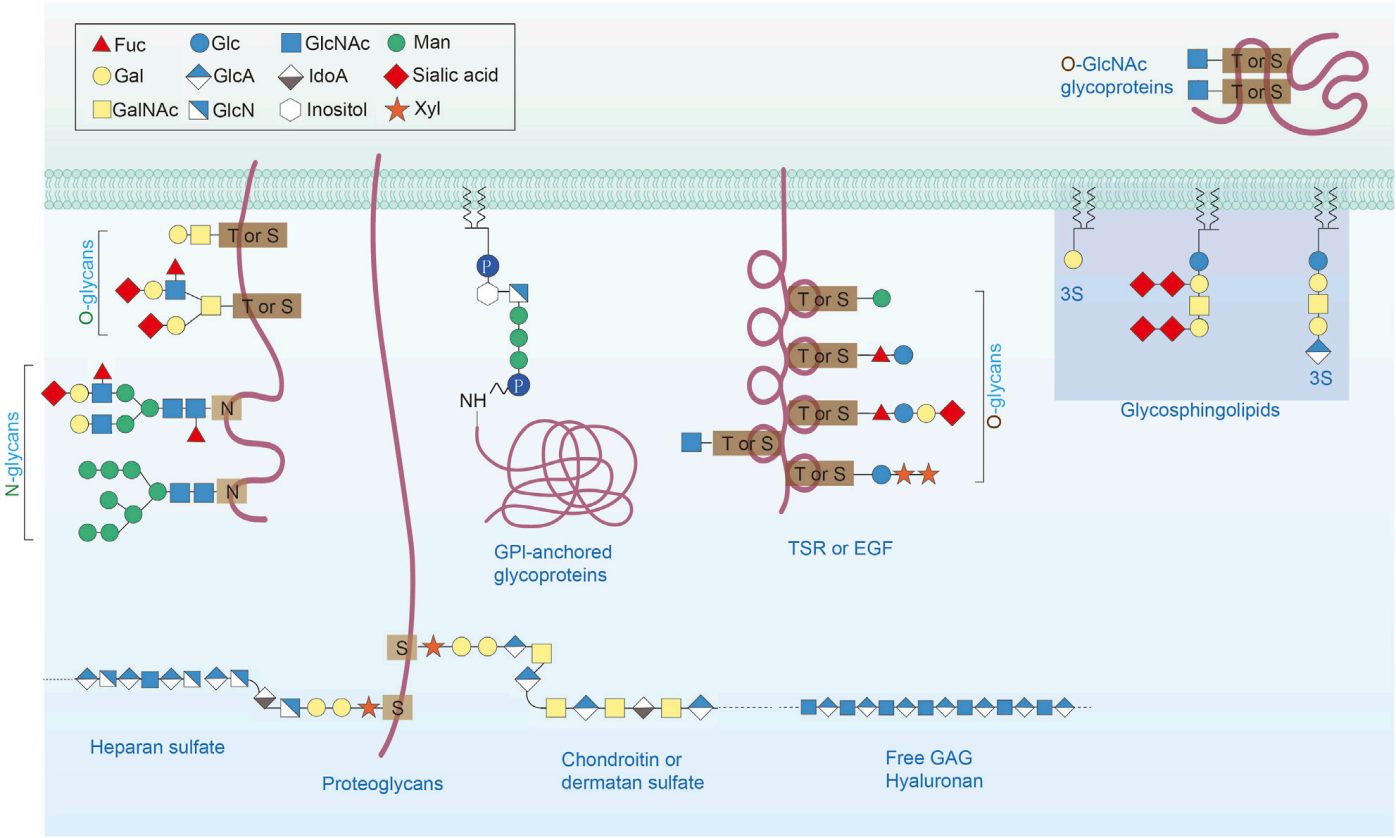
Major types of glycosylation in humans. Protein glycosylation comprises O-linked glycans, N-linked glycans, glycosaminoglycans, phosphorylated glycans and glycosylphosphatidylinositol (GPI) attached to peptide backbones and C-mannosylation to the tryptophan residues.

Protein glycosylation comprises O-linked glycans, N-linked glycans, glycosaminoglycans, phosphorylated glycans and glycosylphosphatidylinositol (GPI) attached to peptide backbones and C-mannosylation to the tryptophan residues (Figure 1) (Hoshi et al., 2024; Dashti et al., 2021). Among these, O-linked β-N-acetylglucosamine glycosylation (O-GlcNAc, also named O-GlcNAcylation), is widely distributed (Loaeza-Reyes et al., 2021). Recently, O-GlcNAc has been increasingly associated with the progression of cardiovascular disorders (Ngoh et al., 2010). Despite the wide acknowledgment of the critical role of protein glycosylation in the modulation of cellular function, our knowledge of the effects of protein glycosylation variation on cardiovascular manifestation is rather inadequate. Here, we completely outline O-linked and N-linked protein glycosylation, as well as intracellular O-GlcNAc protein modification, and discuss how these modifications result in normal function cardiovascular system and their roles in CVDs. Finally, we review the potential function of protein glycosylation in the cardiac and vascular system, highlighting the importance of these issues for future research.

### Overview of protein glycosylation

**FIGURE 2:**
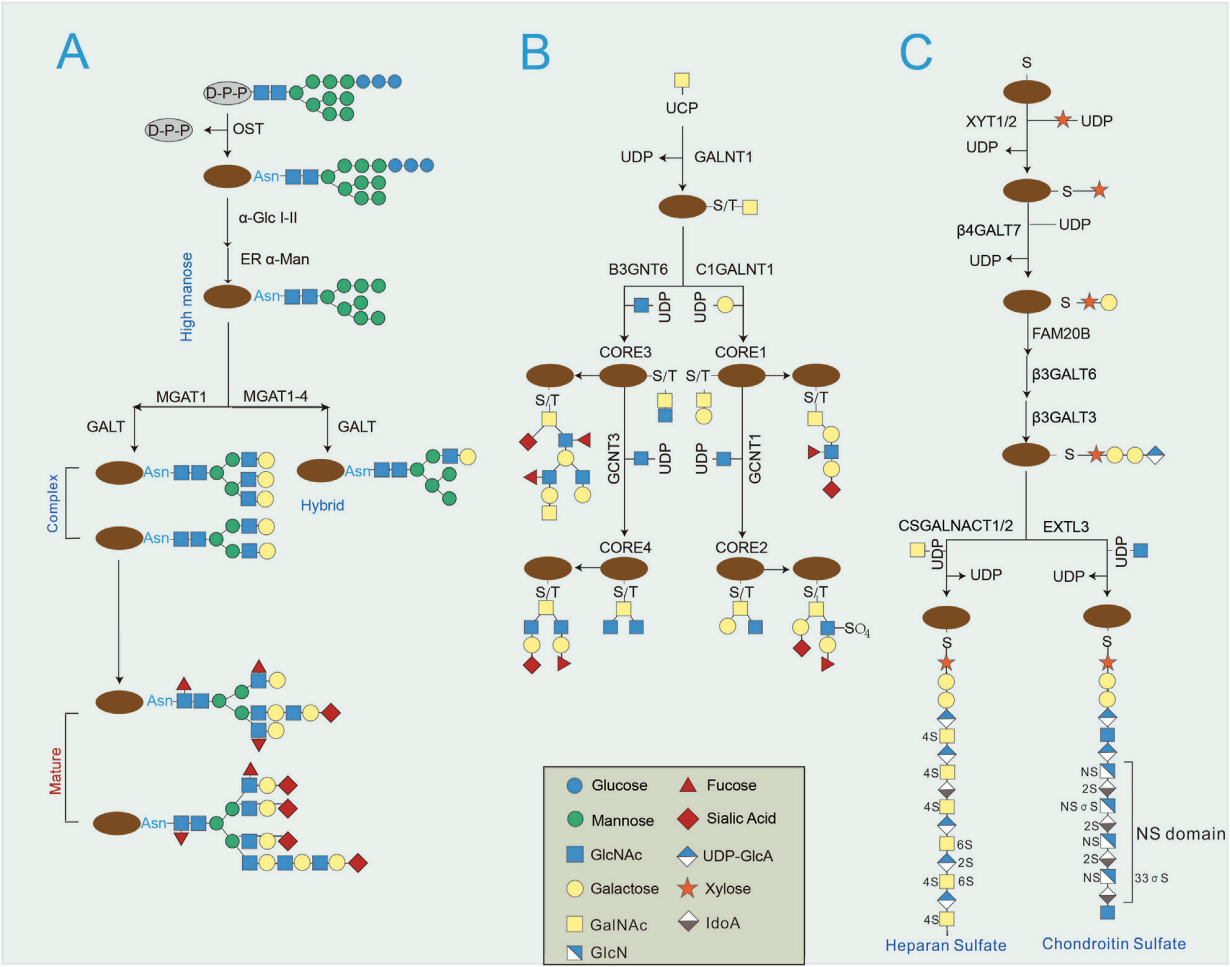
The primary synthesis of N-glycosylation and O-glycosylation. **(A)** N-glycosylation initiates with a preassembled 14 sugar glycan forms dolichol to Asn residues by the Asn-X-Ser/Thr sequence. Subsequently, glycans are further processed by glucosidases, mannosidases and glycosyltransferases, producing hybrid and complicated N-glycans. **(B)** O-glycosylation begins by adding sugar molecule to Ser or Thr residues, followed by various core structure extensions and elongations. **(C)** Proteoglycans belong to a unique group of O-glycosylated types of proteins, which starts by adding xylose to Ser residues by XYLT1/2. Proteoglycans contain glycosaminoglycans in the protein core, which is composed of a common tetrasaccharide linker, Xyl-Gal-Gal-GlcA, forming linear polysaccharides. Afterwards, repeating disaccharide units are sequentially incorporated, such as GlcNAc, GalNAc, GlcA and iduronic acid.

Protein glycosylation is catalyzed by enzymes that covalently attach glycans on the hydroxyl or other functional residues of proteins and is quite different with glycation, which undertakes the conjunction of carbohydrates to certain proteins independent of enzymes (Birukov et al., 2022). Carbohydrates are typically attached either to asparagine (Asn) residues by N linkages or serine (Ser) and threonine (Thr) residues by O linkages in mammals. Other rare forms, including tryptophan on mannose by C-linkage, phosphor (P)-glycosylation and cysteine S-glycosylation, are not addressed in detail in this review (Figure 1) (Majek et al., 2015). The endoplasmic reticulum (ER) and Golgi compartments are the exclusive sites for N-glycosylation and O-glycosylation, except for hyaluronan and O-GlcNAc. In the process of N-glycosylation, a preassembled 14 sugar glycan forms dolichol and is transferred to Asn residues in the Asn-X-Ser/Thr motif, mediated by oligosaccharyltransferase complex (Figure 2). Subsequently, glycans are further processed, producing three types of glycans, according to the processing extent: ‘high mannose’ (minimum processing), ‘hybrid’ and ‘complex’ (maximum processed) (Memarian et al., 2021). The ends of N-glycans are attached to sialic acid, fucose and other monosaccharides, which greatly enriches the variety of glycan types and fulfills additional functional properties, such as protein interactions, protein stabilization, and iron transportation. In contrast, O-glycosylation begins by attaching a single sugar to Ser or Thr residues, and then extensions and elongations of other structures (Liu et al., 2024). To date, O-N-acetylgalactosamine (GalNAc) are the most common O-glycoproteins, which attach to Ser or Thr residues by 20 GalNAc transferases (GALNTs) (Zhang et al., 2024). Similar to N-glycosylation, the ends of O-glycosylation also engage in the attachment of fucose or sialic acid (Figure 2) (Chernykh et al., 2024).

**FIGURE 3:**
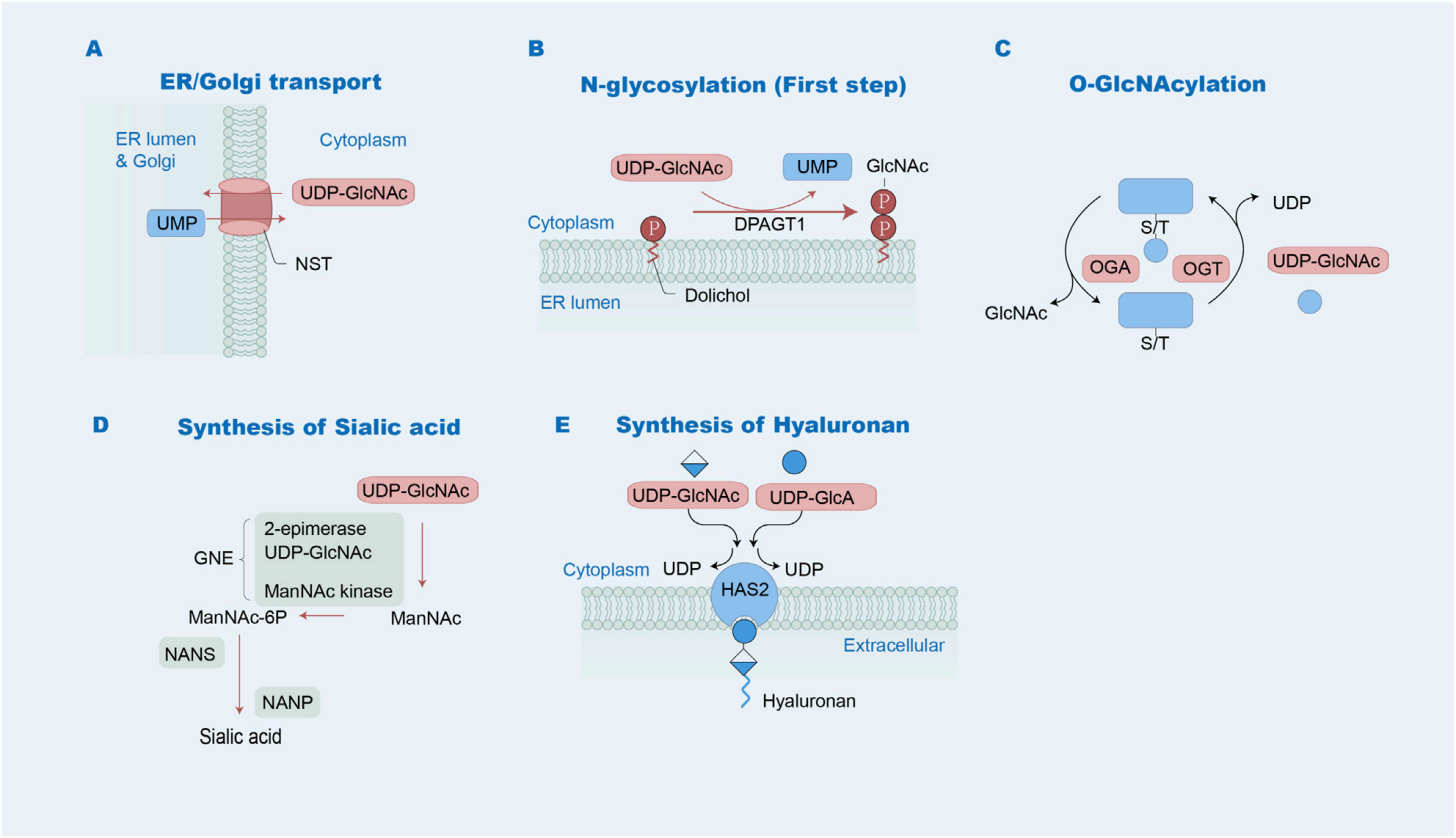
UDP-GlcNAc metabolism pathways. **(A)** Delivery to the Golgi apparatus or ER: UDP-GlcNAc is transported into the Golgi or ER by NSTs, accompanied with the export of UMP. UDP-GlcNAc is available for the production of the N-glycosylation and O-glycosylation of proteins. **(B)** GlcNAc-1-P from UDP-GlcNAc is transferred to Dol-P, forming Dol-P-P-GlcNAc in the initial process of N-glycan synthesis on the ER via DPAGT1. **(C)** UDP-GlcNAc is used as the substrate for the attachment of O-GlcNAc by OGT. **(D)** Sialic acid generation: UDP-GlcNAc is catalyzed by the UDP-N-acetylglucosamine 2-epimerase/N-acetylmannosamine kinase (GNE), followed by N-acetylneuraminic acid synthase (NANS) and N-acylneuraminate-9-phosphatase (NANP) to form sialic acid. **(E)** Hyaluronan synthesis: UDP-GlcNAc and UDP-GlcA are catalyzed by hyaluronan synthases, such as HAS2, with the concomitant release of UDP.

Proteoglycans belong to a unique group of O-glycosylated proteins, which attach xylose to serine residues with the aid of xylosyltransferase 1 (XYLT1) or XYLT2 (Figure 2) (Mongiat et al., 2024). Proteoglycans are associated with the modulation of extracellular matrix (ECM) and can be grouped into four categrories on the basis of their location: extracellular, pericellular intracellular, cell surface, and intracellular. Unlike other glycoproteins, proteoglycans contain glycosaminoglycans in the protein core, which is composed of a common tetrasaccharide linker, Xyl-Gal-Gal-GlcA, forming linear polysaccharides (Melrose, 2024). Afterwards, repeating disaccharide units are sequentially incorporated, such as GlcNAc, GalNAc, glucuronic acid (GlcA) and iduronic acid (Alcaide-Ruggiero et al., 2023). Proteoglycans are critical for ECM regulation and are essential components of myocardial remodeling in the heart (Mongiat et al., 2024). Hyaluronan, a linear glycosaminoglycan polymer that is composed of repetitive disaccharide GlcNAc and GlcA, is similarly associated with ECM regulation and heart remodeling (Han et al., 2024). Hyaluronan synthases (HAS1, HAS2 and HAS3) mediate the formation of hyaluronan at plasma membrane (Figure 3) (Rabelink et al., 2024). In addition, as another class of extracellular glycoproteins, matricellular proteins play important roles in cell and ECM interactions such as signaling, adhesion, proliferation. Matricellular proteins are composed of thrombospondins, secreted protein acidic and rich in cysteine (SPARC) proteins, tenascins, the CCN family of proteins, periostin, and osteopontin (Frangogiannis, 2022). Owing to a lack of glycosaminoglycan motif, most matricellular proteins do not belong to proteoglycans.

Initially, glycoproteins were believed to be either located on the membrane or extracellular proteins; however, in 1984, O-GlcNAc modified proteins within cells were observed and found to be widely distributed within the intracellular compartment (Lunde et al., 2012). Unlike multiple enzymes involved in classical protein glycosylation, the addition and removal of O-GlcNAc from Ser and Thr protein residues is catalyzed by a unique glycosyltransferase, O-GlcNAc transferase (OGT), and a single glycosylhydrolase, O-GlcNAcase (OGA) (Liu et al., 2007).

O-GlcNAc modified proteins remain one of the most enriched types of glycosylation in eukaryotic cells and was initially ignored (Dassanayaka and Jones, 2014). First, the O-GlcNAc modification is typically observed on intracellular proteins. However, Matsuura et al., revealed O-GlcNAc modification on the *Drosophila* sp. Notch receptor, which is extracellular in 2008 (Belke, 1985). Unlike OGT-mediated O-GlcNAcylation, extracellular O-GlcNAc is attached to epidermal growth factor (EGF)-like repeat domains in the ER. This process is catalyzed by a unique glycosyltransferase, EGF-domain-specific OGT (EOGT) (Chatham et al., 2021). Unlike intercellular O-GlcNAcylation, extracellular O-GlcNAc can be further processed by galactose and sialic acid in mammals. EOGT is highly conserved among species and shares little similarity with OGT (Chen et al., 2019).

### The hexosamine biosynthesis pathway

All types of protein glycosylation are initiated by an essential precursor, uridine diphosphate-GlcNAc (UDP-GlcNAc), which is catalyzed by the hexosamine biosynthesis pathway (HBP) (Selitrennikoff et al., 1976). First, fructose-6-phosphate is converted to glucosamine-6-phosphate mediated by glutamine–fructose-6-phosphate aminotransferase (isomerizing) 1 (GFAT). GFAT comprises GFAT1 and GFAT2, encoded by varied genes, as well as a variant (GFAT-L or GFATAlt), which is primarily identified in cardiac and skeletal muscle (Olson et al., 2020). GFAT activity can be phosphorylated regulated and allosterically inhibited by glucosamine-6-phosphate and UDP-GlcNAc. GFAT can also be transcriptionally regulated predominantly in the heart. The functions of GFAT isoforms of are confusing at present; some believe that GFAT1 mainly regulate O-GlcNAc levels in cardiomyocyte, whereas others are convinced that GFAT2 is the main modulator in the heart (Paneque et al., 2023; Filippis et al., 2002). The next step of the HBP is the UDP-GlcNAc synthesis.

### UDP-GlcNAc metabolism

UDP-GlcNAc is a critical component in various glycosylation processes in the ER and Golgi (Jones et al., 2014; Laczy et al., 2011). Nucleotide sugar transporters (NSTs) mediate the transportation of UDP-GlcNAc from the cytosol into these compartments (Figure 3). N-Acetyl-D-glucosamine 1-phosphate (GlcNAc-1P) from UDP-GlcNAc is transferred to dolichol phosphate (Dol-P), forming dolichol pyrophosphate Glc-NAc (Dol-P-P-GlcNAc) in the initial process of N-glycan synthesis on the extracellular lumen of the ER via UDP-GlcNAc–dolichyl-phosphate N-acetyl glucosaminephosphotransferase (DPAGT1) (Figure 3). Additionally, UDP-GlcNAc is necessary for the formation of sialic acid, during which the initial step is facilitated by bifunctional UDP-GlcNAc 2-epimerase/N-acetylmannosamine kinase (GNE). UDP-GlcNAc and UDP-GlcA are necessary for the synthesis of hyaluronan. Finally, UDP-GlcNAc is used as the substrate for attaching O-GlcNAc to proteins (Marshall et al., 2005; Sage et al., 2010).

### Interactions between O-GlcNAc and N- and O-glycosylation

Interactions between O-GlcNAc and classic N-glycosylation or O-glycosylation has been underestimated, probably owing to the different distributions where these protein modifications occur. However, accumulating evidence indicates that O-GlcNAcylation modulates glycan processing to some extent. UDP-GlcNAc transportation into the Golgi is monitored by OGT and O-GlcNAc and therefore impacts the biosynthesis of N-glycan (Marshall et al., 1991; Willems and van Engelen, 2016). Moreover, OGT deletion affected the regulation of N-glycosylation and O-glycosylation (Lee et al., 2021). O-GlcNAcylation is important in the synthesis of hyaluronan. O-GlcNAcylation also modulates GNE activity, an enzyme involved in the synthesis of sialic acid (Lima et al., 2009; Gonzalez-Rellan et al., 2022). Disturbance of O-GlcNAc homeostasis dramatically altered O-glycans but did not change N-glycans in *Caenorhabditis elegans*. Although the crosstalk of these modifications has not been studied in the heart, they are still critical aspects when evaluating changes in O-GlcNAcylation during cardiac (patho)physiology via genetic or pharmacological interventions.

### Heart and vascular function

N-linked and O-linked glycosylation abnormality can lead to multisystem defects, such as congenital disorders of glycosylation (CDGs), a rare and heterogeneous genetic disorders (Boyer et al., 2022). Approximately 20% of the CDGs presented severe cardiac pathologies, including structural abnormalities, cardiomyopathies and arrhythmias, indicating glycosylation importance on cardiovascular function (Pascreau et al., 2023). Many glycosyltransferases and other glycoproteins are embryonically lethal in global knockout mice, indicating the critical role of glycosylation in embryonic development (Greczan et al., 2022). The expression levels of cardiac glycogenes, as well as N-glycans, are considerably different between atrial and ventricular cardiomyocytes, which is quite consistent with that in cardiomyocyte homeostasis (Buyukdogan and Hancer, 2023).

**FIGURE 4:**
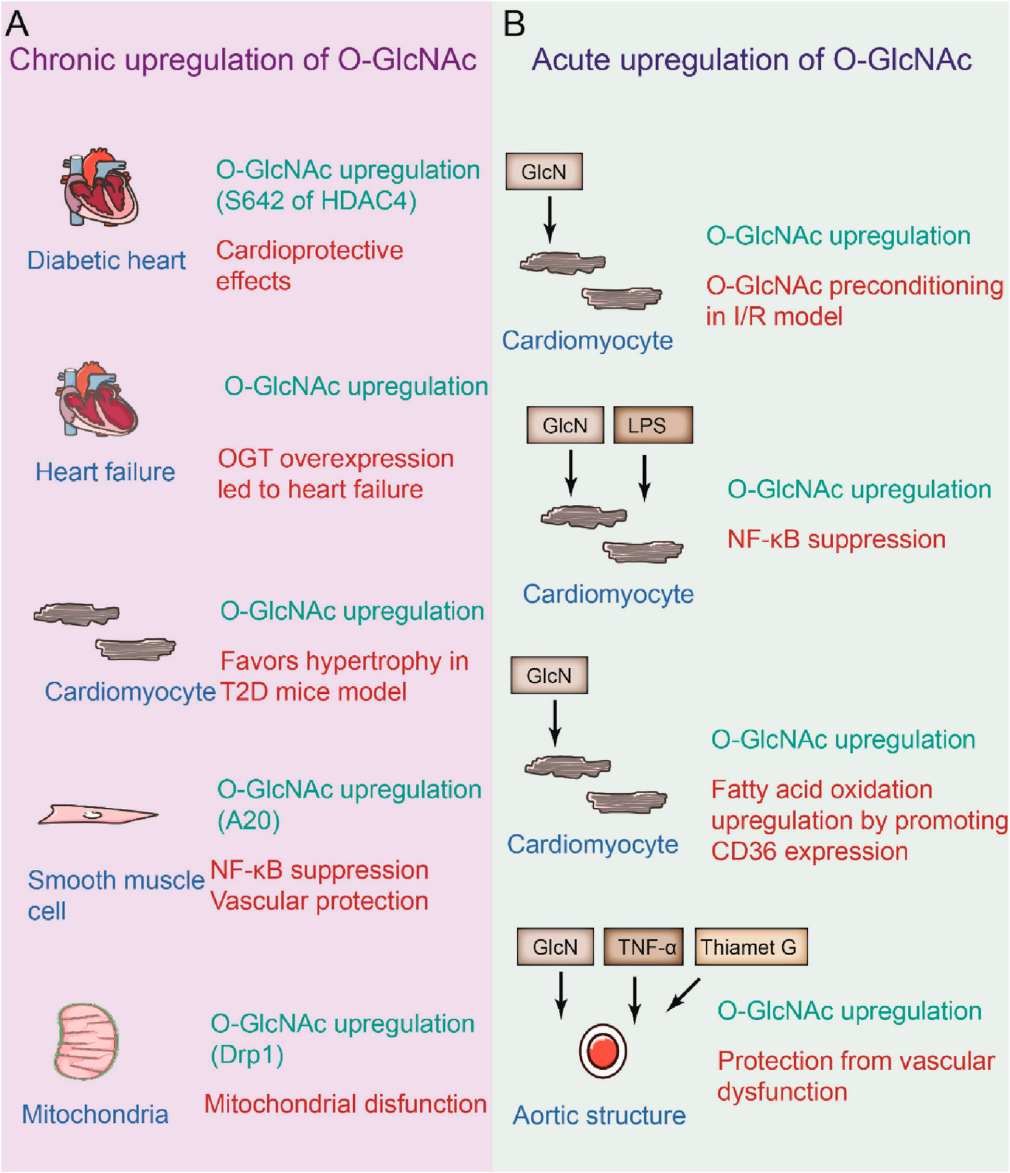
Beneficial and defective effects of O-GlcNAcylation by chronic or acute upregulation of specific modification. **(A)** Under chronic elevated O-GlcNAcylation on diabetic hearts, heart failure, cardiomyocyte, smooth muscle cell. **(B)** Acute elevation of O-GlcNAcylation on cardiomyocyte and aortic segments under certain circumstance.

Analysis of the cardiac glycoproteomes of young (3-month-old) and aged (22-month-old) male mice revealed that the ratio of high-mannose N-glycans increased and that the number of complex N-glycans decreased with age, potentially resulting in a functional decline in the cardiovascular system (Paton et al., 2021). Cardiomyocyte-specific *Ogt* deletion mice presented high perinatal mortality accompanied by defects in cardiac maturation, and in adult animals it was associated with dilated cardiomyopathy, suggesting O-GlcNAcylation is important in the heart (Xiong et al., 2022). *Ogt* flox/flox mice crossed with cardiac troponin T (Tnnt2)-Cre transgenic mice revealed the importance of OGT and O-GlcNAc in early cardiac development via the modulation of angiopoietin 1 expression (Wang H. F. et al., 2023). Conditional overexpression of dominant-negative splice variant of OGA (dnOGA) in cardiomyocytes dramatically elevated cardiac O-GlcNAc levels and subsequently resulted in cardiac hypertrophy, abnormal cardiac remodeling and moderate diastolic impairment after 6 months (Bolanle et al., 2021). In contrast, in mice, OGA specific overexpression in cardiomyocyte had no significant effect on the heart for up to 3 months, although it lowered overall O-GlcNAc levels to a half. During acute cardiac damage, the role of O-GlcNAc, especially after ischemia-reperfusion, was explored. The cardiac level of O-GlcNAc in ischemia-reperfusion was markedly reduced, which is associated with aggravated cardiac injury (Jensen et al., 2019). Conversely, reducing O-GlcNAc levels in cardiomyocyte prevents hypoxia stress in these cells. In contrast, an acute increase in O-GlcNAc levels seems to be cardioprotective, especially in the context of myocardial ischemia-reperfusion injury, accompanied by increased tolerance to ischemia-reperfusion injury in female mice (Figure 4) (Jensen et al., 2019). Therefore, these studies highlight the importance of homeostasis on cardiomyocyte O-GlcNAc levels since rare levels of O-GlcNAcylation make the heart vulnerable to acute injury, whereas persistent increases in O-GlcNAc levels induce chronic injury (Figure 4).

### CVD and cardiac remodeling

Hypertension or valvular disease, heart failure may originate from genetic factor-induced cardiomyopathies, chemotherapy-mediated cardiotoxicities, and multiple causes (Luan et al., 2021a; Luan et al., 2021b; Luan et al., 2024). Specific α-1,3-mannosyl-glycoprotein 2-β-N-acetylglucosaminyl transferase deletion in cardiomyocyte led to reduced complex N-glycans and increased high-mannose N-glycans, leading to the progression of dilated cardiomyopathy and death before maturation (Ednie et al., 2019). Deletion of ST3 β-galactoside α-2,3-sialyltransferase 4 presented dilated cardiomyopathy (Zhu et al., 2024). In a heart failure canine model, the level of N-glycosylation on calsequestrin 2, which is relevant with Ca^2+^ modulation in the sarcoplasmic reticulum (SR), is dramatically reduced, indicating defective Ca^2+^ handling. Overall, the overall variation in complex N-glycans is likely a signature of heart failure and potentially promotes the pathogenesis of this abnormity (Young et al., 2024).

#### Cardiac hypertrophy

Left ventricular hypertrophy is a risking factor in the progression of heart failure, which probably results from increased afterload due to hypertension, aortic stenosis or genetic factors (Wang et al., 2024). In the heart of individuals with cardiac hypertrophy, the mRNA levels of *Gfat* and UDP-GlcNAc are increased in mice and rats, indicating increased substrate availability resulting from protein glycosylation in the hypertrophic heart (Osterholt et al., 2013). Afterwards, a cardiac hypertrophy animal model by pressure overload- and isoprenaline-induced cardiac hypertrophy exhibited increased overall protein N-glycosylation and sialylation levels (Young et al., 2024). However, increased levels of mucin-type O-glycosylated proteins and decreased levels of N-glycosylation were identified in hypertrophy induced by high-salt hypertension (Lunde et al., 2012; Marsh and Dell'Italia, 2011). Additionally, increased O-GlcNAcylation levels have been revealed in hypertrophic rodent and human hearts. Dysregulation of various forms of protein glycosylation are induced in cardiac hypertrophy, accompanied by marked increases in the expression of glycogenes, such as Galnt1, Galnt2 and Galnt7, relevant to initiation of O-glycosylation, as well as decreases in glycosyltransferases associated with core extension (Dozio et al., 2021). Cardiac hypertrophy induces a set of variations in transcription factors in a time-dependent manner. However, how these genes are regulated during this process remains unknown, probably owing to the less studied mechanisms in the regulation of glycogene expression.

Many glycogenes are found epigenetically regulated in various cancers as well as in pathological cardiac hypertrophy, suggesting a possible mechanism of glycogene level in the heart. At present, increased O-GlcNAc levels in both animal and human cardiac hypertrophy is widely acknowledged. Two questions remain unanswered. First, the causative association between increased O-GlcNAc levels and the progression of hypertrophy remains unsolved. Second, the exact role of increased O-GlcNAcylation in cardiac remodeling is still unknown. The classical opinion is that the activation of calcineurin, followed by dephosphorylated nuclear factor of activated T cells (NFAT) and nuclear transfer, initiates hypertrophic transcriptional signaling in cardiomyocytes. The O-GlcNAcylation increase in cardiomyocytes of neonatal rat is adequate and indispensable for increasing transcriptional activity of NFAT and initiating cardiomyocyte hypertrophy (Collins and Chatham, 2020). Another study revealed that stimulation with phenylephrine, a hypertrophic agonist, increases O-GlcNAc levels in cardiomyocytes by increasing GFAT protein levels. In this study, the phosphorylation of GFAT mediated by AMPK reduced O-GlcNAc levels, suggesting that the relief of cardiac hypertrophy by activating AMPK *in vivo* largely contributed to the inhibited increase in O-GlcNAc levels (Zibrova et al., 2017). Both GFAT1 and GFAT2 are involved in adverse cardiac remodeling by mediating O-GlcNAc levels (Ishikita et al., 2021). Notably, the possibility of protein N-glycosylation and O-glycosylation occurring during this process, which is also capable of inducing cardiac remodeling, is not excluded. Cardiomyocyte-specific overexpression of OGT led to increase in O-GlcNAc levels, contributing to dilated cardiomyopathy, ventricular arrhythmias and premature death (Ha et al., 2023). On the contrary, OGA overexpression dramatically relieved both the increased O-GlcNAc level and cardiac remodeling. However, O-GlcNAc levels decrease did not protect against pressure overload-induced cardiac dysfunction before or after surgery. These studies demonstrated that increase in O-GlcNAc levels are capable of inducing cardiac hypertrophy, although this effect may result from other roles of OGT, such as a protease or protein scaffold.

A long-term elevation of cardiomyocyte O-GlcNAc levels (24 weeks) can lead to cardiac hypertrophy (Ha et al., 2023). Although accumulating evidence supports the driving role of elevation in O-GlcNAc levels in pathological hypertrophy and remodeling, the mechanisms underlying how increased O-GlcNAcylation affects cardiac function remain elusive. Increased O-GlcNAcylation can contribute to significant reprogramming of transcription, regardless of the method used (Very et al., 2024). Among these studies, genes associated with mitochondrial oxidative phosphorylation were changed, impairing mitochondrial function. Consistently, the level of O-GlcNAcylated proteins enriched in oxidative phosphorylation was markedly changed in pressure overload-induced hypertrophy, as revealed by a proteomics study. Moreover, alterations in fatty acid and glucose metabolism pathways were also observed. Additionally, the expression of redox signaling proteins also changed, such as significantly elevated NADPH oxidase, in the dnOGA-overexpressing model, similar to AAV-mediated OGT overexpression in mouse hearts (Begum et al., 2022).

#### Diabetic cardiomyopathy

In adverse cardiac remodeling, diabetes acts as an independent risk factor, but its effects on the myocardial glycoproteome are less studied. As a result, how the overall profile of N-glycosylation or O-glycosylation in the heart is altered by diabetes remains unidentified. As shown by a previous study, the levels of N-glycosylated proteins in the hearts of db/db mice (with type 2 diabetes) were globally elevated (Kohda et al., 2009; Ganguly et al., 1990; Zhao et al., 2018). Specifically, α-1,6-fucosylation levels were elevated, with consequences that were not adequately identified. Matricellular proteins are involved in aggravated cardiac fibrosis stimulated by diabetes (Russo and Frangogiannis, 2016). For example, elevated thrombospondin 1 (TSP1) is associated with increased fibrosis in db/db mice. However, TSP1 deletion led to left ventricular dilatation instead of a protective effect (Rogers et al., 2014). Additionally, the activity of TGFβ and SMAD did not change in TSP1-deleted db/db mouse hearts, despite being canonical activators of TGFβ. In contrast, TSP1-deleted db/db mouse presented reduced collagen accumulation and increased matrix metalloproteinase activity in the hearts. The obvious paradox of *Tsp1* deletion in the hearts of mice may be due to systemic deletion instead of cardiomyocyte-specific deletion, which contributes to different effects in other tissues. Decorin overexpression in rats via AAV attenuated cardiac fibrosis and inflammation and restored the contractile function induced by diabetes by attenuating TGFβ1 activity and the nuclear factor-κB (NF-κB) pathway (Chen et al., 2020). Elevation in decorin levels also promoted angiogenesis in diabetic rodent hearts.

The expression of CCN family member 2 (CCN2) was dramatically increased in type 1 diabetes, which was accompanied by increased TGFβ, which was dependent on the protein kinase Cβ (Tsoutsman et al., 2013). Mouse CCN2 deletion in either cardiomyocytes or activated fibroblasts showed that CCN2 in cardiomyocytes promoted only fibrotic remodeling, whereas CCN2 from fibroblasts showed obvious fibrotic features, indicating that CCN2 derived from fibroblasts was involved in diabetes-mediated cardiac remodeling (Gravning et al., 2013).

Diabetes induces increased O-GlcNAc levels in the heart and vascular system, and is involved in contractile dysfunction and adverse remodeling (Chen et al., 2019; Akimoto et al., 2000; Vaidyanathan and Wells, 2014; Cox, 2013). Heart OGA overexpression lowered O-GlcNAc levels and relieved contractile function in type 1 diabetes mouse model (Akimoto et al., 2000). In addition, the beneficial effect of reduced O-GlcNAc levels was related to the improvement in ATPase 2a levels, which restored Ca^2+^ reuptake into the SR and ameliorated the impacts of diabetes on relaxation. OGA overexpression in the heart relieves left ventricular diastolic dysfunction and adverse remodeling in type 2 diabetes (Kaleem et al., 2021). These phenotypes are closely associated with cardiac phosphoinositide 3-kinase-Akt signaling. The underlying mechanisms can be largely attributed to increased O-GlcNAc levels. Increased O-GlcNAcylation is linked to impaired role of contractile proteins, such as actin, myosin and troponin (Jo et al., 2023). The unfavorable outcome of diabetes on the heart are associated with metabolic dysfunction, including abnormal mitochondrial function. Multiple mitochondrial proteins have been found to be modified via O-GlcNAcylation. Increased cardiac O-GlcNAc levels mediated by diabetes are also related to mitochondrial fragmentation and mitochondrial DNA damage (Chen et al., 2019). Among those, Calcium–calmodulin (CaM)-dependent protein kinase II (CaMKII) is believed to mediate the adverse consequence of diabetes on the heart, and its O-GlcNAcylation is increased in human diabetic hearts, accompanied by increased kinase activity. The increase in CaMKII activity mediated by O-GlcNAcylation is potentially involved in the pathogenesis of arrhythmias and is also responsible for increased production of reactive oxygen species in cardiomyocytes. Notably, the increase in CaMKII signaling can be neutralized by O-GlcNAcylation on HDAC4 in diabetic mouse models (Erickson et al., 2013). Moreover, autophagic signaling may also be involved in O-GlcNAc-mediated cardiac dysfunction, which has already been confirmed in animal models of type 1 or type 2 diabetes (Marsh et al., 2013). A rising number of autophagic regulatory proteins have been revealed to be potential O-GlcNAc targets. Furthermore, circadian proteins, which are O-GlcNAcylated, are also altered in the hearts of diabetic model mice (Hui et al., 2024). Interestingly, cardiac O-GlcNAc levels are constantly changing throughout the day, and once the cardiac circadian clock is disrupted, these changes disappear in the heart. Therefore, the regulation of circadian-related pathways has emerged as a critical factor in diabetic cardiac remodeling.

Diabetic cardiomyopathy is often associated with myocardial fibrosis. Previous research has shown that both cardiac fibrosis and protein O-glycosylation are heightened in diabetes. Prolonged exposure to high glucose levels significantly upregulated type III collagen expression in rat cardiac fibroblasts (RCFs), yet had no impact on protein O-glycosylation. Treatment with glucosamine not only elevated the expression of collagen types I and III, but also augmented O-glycosylated proteins. These findings indicate that HBP activation-induced protein O-glycosylation modulates collagen expression and may contribute to the development of diabetic cardiomyopathy. Understanding how O-GlcNAcylation contributes to these changes can provide insights into the underlying mechanisms and potential therapeutic targets.

Myocardial fibrosis is known as an adverse consequence of diabetes; however, the underlying mechanisms remain poorly understood. One generally accepted explanation is that hyperglycemia activates cardiac fibroblasts, inducing myofibroblast proliferation and ultimately leading to increased ECM deposition via oxidative stress or proinflammatory pathways (Feng et al., 2020). A short-term increase in cardiomyocyte O-GlcNAc levels promoted profibrotic gene expression, and long-term exposure induced fibrosis, along with increased ECM structure and collagen synthesis pathways (Wang J. et al., 2023; Yokoe et al., 2023). In all, O-GlcNAcylation level in cardiomyocyte may directly result in ECM remodeling and that excess O-GlcNAc accumulation in cardiomyocytes may act as a factor in aggravating fibrosis.

#### Vascular disease

Vascular inflammation is characterized by the enrichment in immune cells on the endothelium, leading to endothelial dysfunction and smooth muscle cell proliferation and remodeling, eventually contributing to vascular diseases, such as atherosclerosis and coronary artery disease (Masbuchin et al., 2021). Multiple cell types and proteins participate in the process, but the potential involvement of O-GlcNAcylation, O-glycosylation and N-glycosylation in this disease remains poorly understood. During the initiation and progression of atherosclerotic lesions, the adhesion of monocytes to the endothelium is fundamental. For example, the level of high-mannose N-glycan modification on the endothelium plays an important role in recruiting proinflammatory monocytes to induce lesions (Herzog et al., 2014). Alterations in high-mannose structures did not disturb neutrophil binding to endothelial cells, highlighting the notion that distinct types of glycoprotein regulation, as well as the expression of adhesion molecules, might act as key regulators in monitoring how immune cell subtypes are recruited to their specific vascular bed when stimulated by stimuli (Chandler et al., 2019). Thus, glycans on the cell surface work as a ‘postcode’, regulating leukocyte trafficking. Moreover, infections can promote the clearance of glycoproteins from vascular cells.

Cardiac fibroblasts interact closely with endothelial cells, influencing vascular homeostasis and function. They can secrete factors such as vascular endothelial growth factor (VEGF) and fibroblast growth factor (FGF), which are essential for angiogenesis and the maintenance of vascular integrity. In pathological states, such as myocarditis, endothelial cells can become activated, leading to increased expression of adhesion molecules and the recruitment of immune cells, which can further exacerbate cardiac injury. Cardiac fibroblasts are exposed to various stressors, including mechanical stress, oxidative stress, and metabolic changes. O-GlcNAcylation can help these cells adapt to stress by modulating stress response pathways. For example, it can influence the activity of heat shock proteins and other molecular chaperones, which are essential for maintaining protein homeostasis under stress conditions.

Metabolic pathways play crucial roles in the processes of atherosclerosis and coronary artery disease, highlighting the importance of O-GlcNAcylation in vascular disease (Hou et al., 2023). At present, increasing studies suggest that increased O-GlcNAc levels are closely linked to the pathophysiology of atherosclerotic disease and suggest that O-GlcNAc modifications may lead to adverse effects following vascular interventions (Miguez et al., 2018). For example, specific *Ogt* deletion in vascular smooth muscle cells (VSMCs) protected against atherosclerosis in a high-fat diet or *Apoe* ^−/−^ mouse model. VSMC-specific *Ogt* deletion induced arterial stiffness, supporting its role in controlling contractility (Hayakawa, 1991). Increased O-GlcNAcylation is found to modulate vascular contractile responses (Marsh and Collins, 2014). Therapies targeting atherosclerosis also led to reduced O-GlcNAc levels (Peretz et al., 1992). In contrast, an acute increase in O-GlcNAc in VSMCs protected against VSMC dysfunction and neointima formation following balloon injury (Hilgers et al., 2012). The initial increase in O-GlcNAc levels may be vascular protective; however, chronic elevation may be detrimental, leading to chronic disease.

## Outlook

**FIGURE 5:**
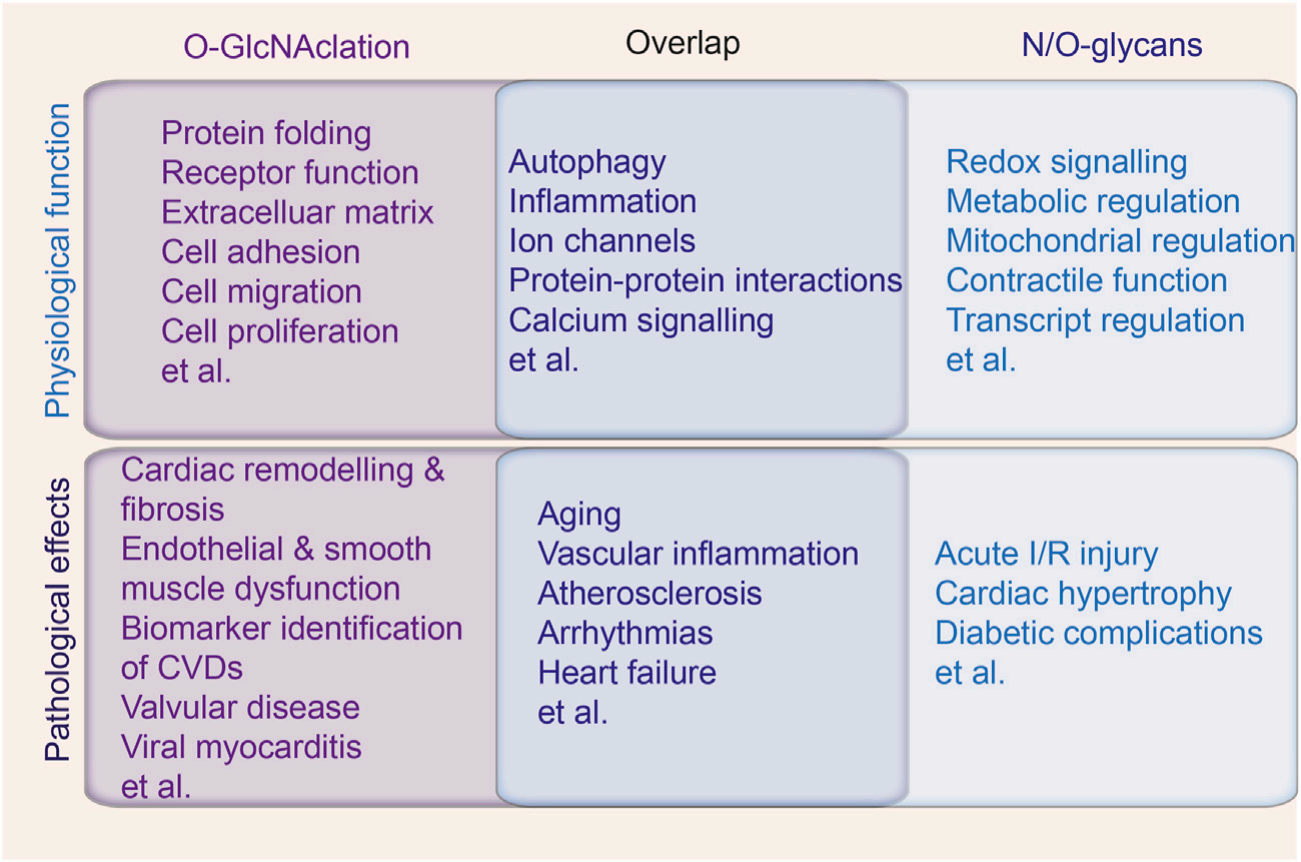
The intersection of N-glycans, O-glycans and O-GlcNAcylation in cardiac physiology and pathology. The physiological function and pathological effects of N-glycans, O-glycans and O-GlcNAcylation in cardiovascular system, with the middle overlap indicating the biological processes and diseases mediated by all the types of protein glycosylation.

Protein glycosylation, particularly O-GlcNAcylation, represents a pivotal PTM that critically regulates cardiac and vascular functions by modulating protein stability, localization, and interactions. Dysregulated glycosylation has been implicated in the pathogenesis of CVDs, contributing to processes such as adverse cardiac remodeling, mitochondrial dysfunction, and vascular inflammation (Figure 5). Despite its recognized significance, the clinical application of glycosylation-related findings remains limited. Elucidating specific glycosylation signatures holds promise for the development of novel biomarkers for early diagnosis and risk stratification, especially in populations predisposed to CVDs, such as individuals with diabetes. Moreover, therapeutic strategies targeting glycosylation pathways, including precise modulation of OGT and OGA activity, present an emerging avenue for intervention. Translational research, supported by advanced tools for glycosylation profiling and mechanistic studies, is essential to bridge the gap between molecular insights and clinical implementation, thereby advancing the field of precision cardiovascular medicine.

Modulating glycosylation pathways, particularly O-GlcNAcylation, represents a promising therapeutic avenue for CVD. Recent findings highlight its dual role in cellular stress responses, offering cardioprotection in acute injury but contributing to chronic pathologies, such as cardiac hypertrophy and diabetic cardiomyopathy. The precise modulation of OGT or OGA activity has the potential to restore glycosylation homeostasis, addressing key mechanisms underlying mitochondrial dysfunction, calcium dysregulation, and inflammation. Advances in small-molecule inhibitors and glycan-targeting therapies provide novel opportunities for intervention.

The therapeutic translation of glycosylation research is hindered by the complexity and context dependence of glycosylations. The tissue-specific roles of glycosylation and the interconnected nature of its pathways pose significant risks of off-target effects, as interventions targeting enzymes such as OGT or OGA may disrupt systemic homeostasis. Additionally, the dynamic and reversible nature of glycosylation demands precise temporal and spatial modulation, which current therapeutic strategies lack. Addressing these challenges requires the development of highly selective modulators, tissue-targeted delivery systems, and predictive models to minimize adverse effects, ensuring safe and effective clinical application.

Mechanistic studies are essential to clarify how glycosylation impacts mitochondrial dysfunction and metabolic regulation in cardiovascular diseases, offering potential therapeutic targets. The development of tools for real-time monitoring of glycosylation during cardiovascular events could further enhance understanding and guide precise interventions.

Glycosylation biomarkers hold significant potential for personalizing treatment strategies by enabling precise risk stratification and monitoring disease progression, particularly in complex conditions such as diabetes-related CVD. These biomarkers could guide tailored interventions, identifying patients who would benefit most from glycosylation-modulating therapies. Furthermore, integrating glycosylation-targeting approaches with existing treatments, such as metabolic or anti-inflammatory therapies, could increase treatment efficacy by addressing the underlying molecular mechanisms involved. This synergy underscores the need for interdisciplinary efforts to validate glycosylation biomarkers and optimize combination treatment strategies for specific patient subgroups.

In summary, targeting glycosylation pathways offers a promising approach for improving cardiovascular health by addressing key mechanisms underlying disease progression, such as mitochondrial dysfunction, inflammation, and metabolic dysregulation. Advancing these therapies requires close collaboration between basic researchers and clinicians to translate molecular insights into effective, patient-centered interventions, bridging the gap between laboratory findings and clinical application.
